# Trait food craving predicts functional connectivity between dopaminergic midbrain and the fusiform food area during eating imagery

**DOI:** 10.3389/fpsyt.2024.1396376

**Published:** 2024-05-07

**Authors:** Francantonio Devoto, Marika Mariano, Edoardo Gornetti, Eraldo Paulesu, Laura Zapparoli

**Affiliations:** ^1^ Psychology Department and NeuroMi – Milan Centre for Neuroscience, University of Milano-Bicocca, Milan, Italy; ^2^ fMRI Unit, IRCCS Orthopedic Institute Galeazzi, Milan, Italy

**Keywords:** food craving, fMRI, ventral tegmental area (VTA), mental imagery, functional connectivity

## Abstract

Neurofunctional coupling between the dopaminergic midbrain (i.e., ventral tegmental area, VTA) and higher-order visual regions may contribute to food craving, leading to the onset or maintenance of obesity. We recently showed that the VTA resting-state functional connectivity with the occipitotemporal cortex, at the level of the fusiform gyrus (FFG), was specifically associated with trait food craving and the implicit bias for food images, suggesting that VTA-FFG connectivity may reflect the association between the visual representations of food and its motivational properties. To further test this hypothesis, this time we studied task-based functional connectivity in twenty-eight healthy-weight participants while imagining eating their most liked high-calorie (HC) or least liked low-calorie food (LC) or drinking water (control condition). Trait food craving scores were used to predict changes in task-based functional connectivity of the VTA during imagery of HC compared to LC foods (relative to the control condition). Trait food craving was positively associated with the functional connectivity of the VTA with the left FFG: people with higher trait food craving scores show stronger VTA-FFG connectivity, specifically for the imagery of the liked HC foods. This association was not linked to the quality of imagery nor to state measures of craving, appetite, or thirst. These findings emphasize the contribution of the functional coupling between dopaminergic midbrain and higher-order visual regions to food craving, suggesting a neurofunctional mechanism by which the mental representations of the HC food we like can become much more salient if not irresistible.

## 1 Introduction

Intense desires to eat a particular food, namely food craving (1), can arise even in the absence of homeostatic energy needs (2) and may dissociate from the circadian fluctuations of hunger (3). Behaviorally, craving for food is assessed with self-report questionnaires addressing state and trait food craving. Measures of *state craving* assess a momentary craving and usually do not differ between people with and without eating disorders, while *trait food* craving quantifies more stable and habitual cravings, typically higher in individuals with eating disorders or obesity (see (4) for a review).

Food craving usually occurs for hyper-palatable and high-calorie foods, such as sweets and fat foods, and it is associated with more frequent food-related thoughts and intake of those foods (5, 6). Accordingly, people who are overweight or obese report higher and more frequent cravings for high-calorie foods compared to normal-weight participants (5, 7), suggesting that the construct taps into relevant clinical aspects of eating behavior (8).

### 1.1 Neurobiology of craving

At the neurobiological level, food craving has been associated with increased activity of the mesocorticolimbic or reward system, a network that is chiefly involved in the motivation to pursue biological and non-biological rewards (9). The reward system comprises the main targets of the dopaminergic projections arising from the ventral tegmental area (VTA) and includes the ventral striatum, hippocampus, amygdala, and orbitofrontal cortex (OFC). Using the “cue-reactivity” paradigm, an experimental task that consists of the exposure to, or imagery of, food-related stimuli (10–13), functional magnetic resonance imaging (fMRI) studies showed that craving for liked food cues is mediated by structures of the reward circuit, including the hippocampus and the dorsal striatum, in addition to the insula (11).

Only a few studies investigated the direct association between brain reactivity to food cues and self-report measures of craving. Using a food/non-food visual discrimination task in a sample of normal-to-obese males, Ulrich and colleagues correlated the brain activity in response to high-calorie *vs* low-calorie food pictures with scores of the state and trait versions of the Food Cravings Questionnaire (14), one of the most widely used self-report scales for the assessment of food craving (15, 16). Though no significant association was found for state food craving, trait food craving was associated with increased activity of the striatum and middle-lateral OFC in response to high-calorie *vs*. low-calorie foods (16).

In healthy-weight males, Chen and colleagues used regional homogeneity to measure local synchronization in resting-state fMRI data to study the neurofunctional correlates of trait food craving (17). The authors found a positive association between trait food craving and regional homogeneity in the bilateral parahippocampal and fusiform gyrus (17).

These previous findings suggest that food craving can be encoded not only in areas involved in reward processing and emotional memory but also in brain regions involved in higher-order visual processing.

### 1.2 Highly selective response to food in the visual cortex

Dovetailing with their biological significance, visual food stimuli are processed by specialized cortical patches in the ventral occipitotemporal cortex, including the fusiform gyrus (FFG) (18–20). Jain et al. (19) identified two strips of the ventral occipitotemporal cortex surrounding the fusiform face area that respond selectively to visual food stimuli (19), here labelled as fusiform food area (FFoA), aligning with the evidence that discrete portions of the ventral visual pathway encode different ecologically relevant stimuli such as faces (21), bodies (22), places (23), and words (24). These results are in line with meta-analytical studies showing that food compared to non-food pictures elicit an extensive activation of the visual system, particularly in the FFG (25–27).

We recently observed that patients with obesity, compared to normal-weight controls, display greater functional connectivity of the VTA with the putative FFoA bilaterally, in addition to decreased connectivity with the left inferior frontal cortex (28). The strength of the functional connections of the VTA with the left FFoA was associated with trait food craving, while connectivity with the inferior frontal cortex was negatively associated with trait food craving scores. VTA-FFoA connectivity was also positively correlated with the implicit bias for visual food stimuli [assessed with the Implicit Association Test (29)], but not with the bias for non-food stimuli (28).

These results led to the hypothesis that VTA-FFoA connectivity may reflect cue-reward associations specific to food stimuli (28) and were interpreted as reflecting an imbalance between brain systems involved in motivational reactivity and those involved in the regulation of the responses to food cues (30). However, brain connectivity was assessed at rest, while participants were not involved in any sensory or cognitive food-related processing.

To make this functional association more explicit, here we used a food cue-reactivity imagery paradigm and assessed whether the VTA-FFoA functional coupling might be specifically seen during imagery of liked high-calorie palatable foods in association with trait food craving.

### 1.3 Aims and predictions

To investigate the relationship between trait food craving and the neural correlates of internally induced craving guided by food cues, we used a food imagery paradigm and studied whole-brain patterns of VTA functional connectivity in a sample of healthy-weight subjects with varying levels of trait food cravings.

Prompted by a visual cue, participants imagined eating their most liked high-calorie (HC) or disliked low-calorie (LC) foods or drinking a glass of water (control condition). We first identified the brain networks generally involved in the imagery process and those specific to the imagery of food (liked HC, disliked LC) compared with the control condition. Then, trait food craving scores were used to predict the differences in VTA whole-brain functional connectivity during imagery of HC compared to LC foods.

In line with previous literature on mental imagery (31–33), we expected significant activity of a fronto-parietal-occipital network during imagery of food and water. We further expected increased activity of reward-related regions, including the mesolimbic system and the orbitofrontal cortex, during imagery of food compared to water.

We also hypothesized a positive association between trait food craving and VTA-FFoA connectivity (30), particularly during imagery of HC compared to LC foods: this would suggest a stronger cue-reward association in subjects with higher food craving. A complete replication of our previous findings would anticipate a negative association between food craving scores and VTA functional connectivity with the inferior frontal cortex. In keeping with previous findings (16, 17), one could also anticipate a positive association between food craving and connectivity of the VTA with the main targets of its dopaminergic projections (e.g., ventral striatum, parahippocampal gyrus).

## 2 Materials and methods

### 2.1 Participants

Twenty-eight healthy adult participants (male/female ratio: 14/14; mean age: 24 ± 4.1 years; mean education level: 15 ± 2.1 years; mean BMI: 21.2 ± 2 kg/m^2^ [range: 18.4–24.9 kg/m^2^]), participated in the study. All the participants were right-handed, as assessed by the Edinburgh handedness inventory (34). The study protocol was approved by the local Ethics Committee (IRCCS San Raffaele of Milan; Prot. CONSUME, 58/INT/2020), and informed written consent was obtained from all subjects. See the **Supplementary Information** and **Supplementary Table S1** for further details on the participants included in the study.

### 2.2 Behavioral measures

Individual food preferences were assessed before the experiment by asking participants to report their liking rating on a 6-point Likert scale ranging from 0 (I don’t like it at all) to 6 (I extremely like it) for 100 foods.

Trait and state food craving were assessed with the Food Cravings Questionnaires (FCQ) (14, 35), whereas hunger and thirst were assessed by means of 100-mm visual analogue scales (VAS).

See the **Supplementary Information** for further details on stimuli selection and the behavioral measures included in the study.

### 2.3 Food cue-reactivity imagery paradigm

fMRI scanning was performed during a food cue-reactivity paradigm involving visually guided mental imagery. Participants were instructed to imagine eating the food (most liked HC food, least liked LC food) or to imagine drinking a glass of water, depending on the picture displayed on the screen. Subjects were instructed to focus on the multisensory experience of eating the food, including its smell, taste, and texture experienced in the mouth. At the end of the task, participants rated the quality of their imagery performance separately for HC, LC foods and water by means of 100-mm VAS ranging from 0 (extremely bad) to 100 (extremely good).

Self-report imagery quality data of the three different conditions (HC, LC, W) were compared by means of a repeated measures ANOVA.

See the **Supplementary Information** for further details on task structure and analytical plan of imagery quality data.

### 2.4 fMRI data acquisition

MRI scans were performed using a 1.5 T Siemens Avanto scanner, equipped with gradient-echo echo- planar imaging (flip angle 90°, TE=40 msec, TR=2000 msec, FOV=250 mm and matrix=64x64). The overall number of the fMRI volumes collected was 260. The first 15 volumes of each sequence (corresponding to presentation of the instructions) were discarded from the analyses.

Subjects were also scanned with an MPRAGE high-resolution T1-weighted volumetric scan for further visualization of the results (flip angle = 35°, TE = 5 ms, TR = 21 ms, FOV = 250 mm, matrix = 256 x 256, TI = 768, for a total of 160 slices with 1 x 1 x 1 mm voxels).

For task functional activation and connectivity analyses, cluster-level inferences were based on parametric statistics from Gaussian Random Field theory (36). Results were thresholded using a combination of a voxel-level p < 0.001 uncorrected threshold, and a familywise corrected p-FWE < 0.05 cluster-size threshold across the whole brain (37).

#### 2.4.1 Task functional activation analyses

A complete description of preprocessing, first-level, and second-level analyses is reported in the **Supplementary Information** **.** At the first level we characterized the brain activity recorded between the appearance and disappearance of the food or water’s pictures, namely the entire imagery process. For each participant, we considered an implicit baseline leading to four contrast images: (i) HC food, (ii) LC food, (ii) water after HC food (W_HC_), and (iv) water after LC food (W_LC_).

In a second-level full-factorial random-effect analysis, two contrasts were computed in order to (i) identify the neural networks commonly involved in mental imagery of food and water (food ∩ water) and (ii) distinguish the brain areas more active during the imagery of food compared to water (food > water).

#### 2.4.2 Task functional connectivity analyses

A full description of preprocessing, denoising, first-level, and second-level analyses is reported in the **Supplementary Information**. After preprocessing and denoising, at first-level seed-based functional connectivity maps were estimated characterizing the spatial pattern of functional connectivity with the seed area, as defined by the bilateral VTA of the AAL3 template (38).

At second-level, linear regressions were performed with trait food craving scores as predictor of the differences in whole-brain functional connectivity of the VTA during the following contrasts:

- [HC > W_HC_] > [LC > W_LC_]. To identify the brain areas whose connectivity with the VTA during imagery of HC compared to LC foods is positively associated with trait food craving scores;
- [LC > W_LC_] > [HC > W_HC_]. To identify the brain areas whose connectivity with the VTA during imagery of LC compared to HC foods is positively associated with trait food craving scores.

## 3 Results

### 3.1 Quality of imagery during fMRI task

The Friedman test showed a significant difference in average ratings of imagery quality across stimuli (χ^2^(2)=21.4, p <.001, Kendall W = .43): the quality of imagery was different across HC, LC foods, and water (**Figure S3** in **Supplementary Information**). Bonferroni-corrected *post-hoc* comparisons showed that participants imagined better HC foods (mean=76.2, SD=19.5) compared to LC foods (mean=48.2, SD=18.3; Wilcoxon W=307, p_bonf_<.001) and water (mean=55.4, SD=23.7; Wilcoxon W=275, p_bonf_=.008). The difference in average imagery quality between LC foods and water was not significant (Wilcoxon W=111, p_bonf_=.51).

### 3.2 Task functional activation results

#### 3.2.1 Conjunction analysis [FOOD ∩ WATER]

**Figure 1:**
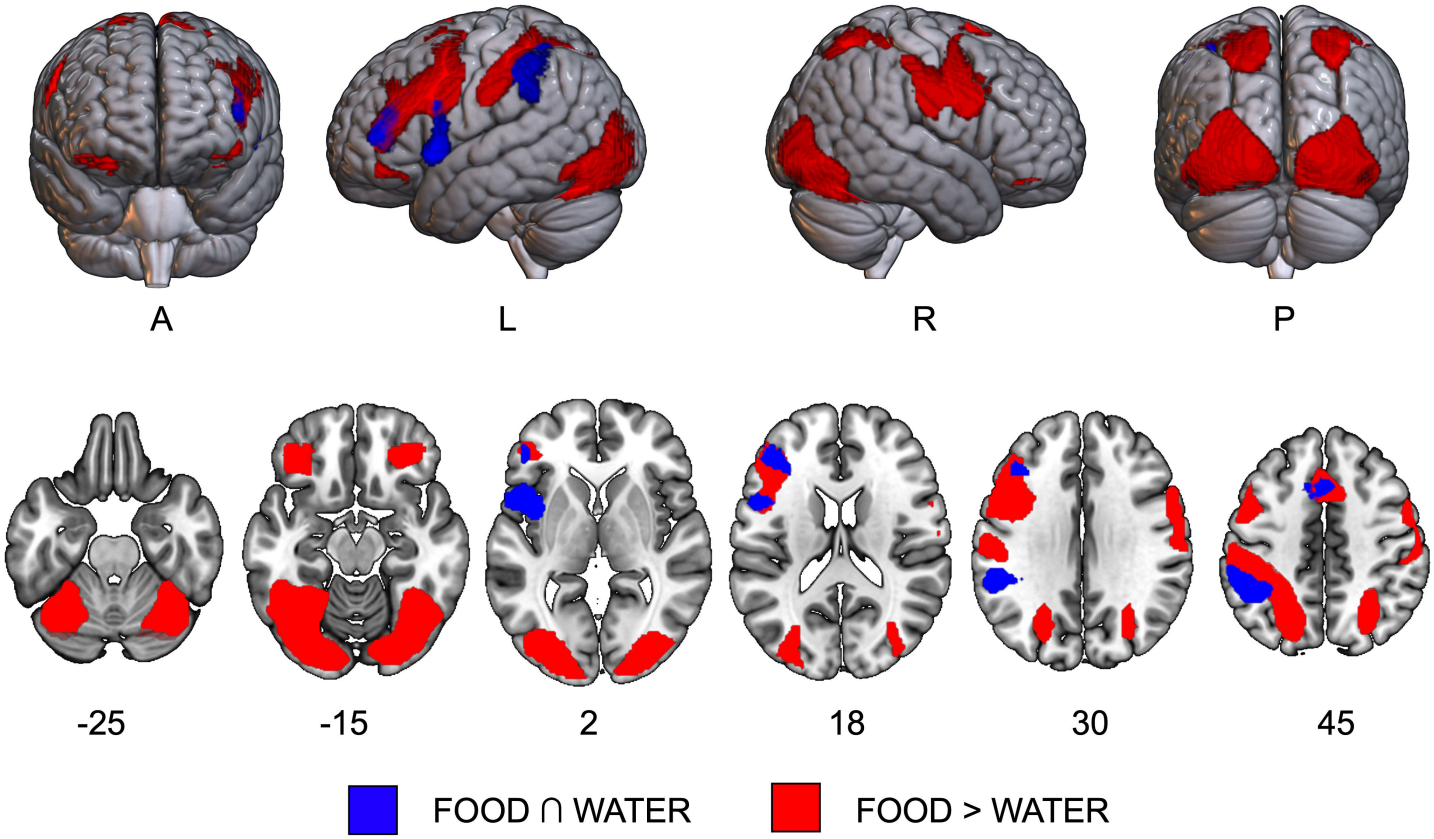
Results of task activation data. The conjunction effect [FOOD ∩ WATER, in blue] and the direct contrast [FOOD > WATER, in red] are overlaid on axial slices of the MNI template. A, anterior view; L, left hemisphere; P, posterior view; R, right hemisphere.

The conjunction analysis revealed a left-lateralized fronto-parietal network comprising the supplementary motor area (SMA), the left inferior frontal and parietal cortex, and the left insula (**Supplementary Table S3A** and **Figure 1**, in blue).

#### 3.2.2 Effect of food over water [FOOD > WATER]

A largely bilateral network was significantly more active during the imagery of food compared to water and included significant clusters of activation in bilateral orbito-frontal, precentral and postcentral gyri, in left supplementary motor area and parietal gyri, and in occipito-temporal areas, including superior, middle and inferior occipital gyri, and right inferior temporal and fusiform gyri (**Supplementary Table S3B** and **Figure 1**, in red).

### 3.3 Task functional connectivity results

#### 3.3.1 Imagery of liked HC compared to disliked LC foods ([HC > W_HC_] > [LC > W_LC_])

**Figure 2:**
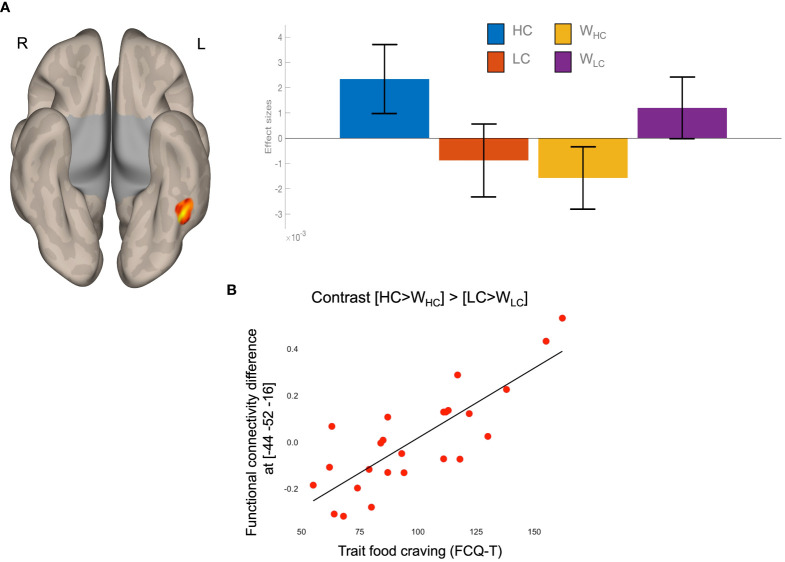
Results of task functional connectivity analyses. **(A)** The left FFG cluster rendered on a semi-flattened cortical surface (inferior view) and effect sizes (mean and standard error) of the food-by-water interaction. **(B)** Scatterplot of the linear association between food-by-water interaction contrast estimated and trait food craving (FCQ-T scores). HC, high-calorie; LC, low-calorie; L, left; R, right; W, water.

The food-by-water interaction on FC values between the VTA and the left fusiform gyrus (FFG) was positively associated with FCQ-T scores (**Figure 2A** and **Table 1**). Higher food craving scores were associated with greater VTA-FFG functional connectivity differences during imagery of HC compared to LC foods (**Figure 2B**).

**Table 1:** Results of task functional connectivity analyses.

	Left hemisphere				Right hemisphere			
Anatomical label (Brodmann area)	X	Y	Z	Z-score	X	Y	Z	Z-score
Fusiform gyrus (37)	-44	-52	-16	4.99*				
	-40	-52	-10	4.63*				

The anatomical label according to the AAL3 template (38) (Brodmann area), coordinates in MNI space, and Z-score of the resulting clusters are reported. *, p <.05 FDR corrected at peak-level.

#### 3.3.2 Imagery of disliked LC compared to liked HC foods ([HC > W_HC_] > [LC > W_LC_])

No significant clusters associated with FCQ-T scores.

#### 3.3.3 Anatomical overlap of the FFG local maxima compared to previous literature

**Figure 3:**
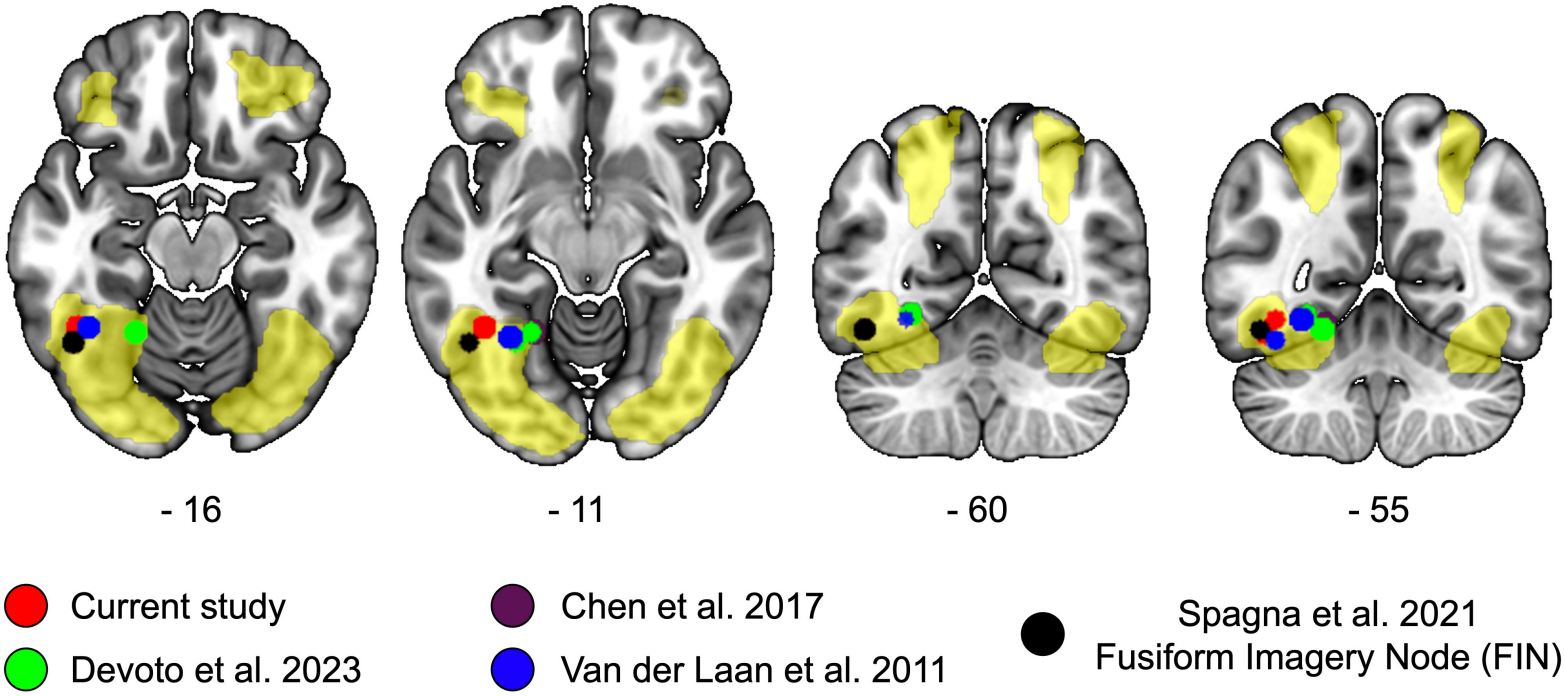
Local maxima of studies on food perception, imagery, and food craving in the fusiform region. We overlaid on a MNI anatomical template the brain regions showing a significant activation in response to food as compared to water (yellow overlay). The local maxima reported in the current study are represented in red, those of our previous resting-state functional connectivity study in green (28); local maxima showing a significant positive association between regional homogeneity and trait food craving is represented in violet (17) [overlapping with (28)]; local maxima associated with the perception of food according to the meta-analysis by Van der Laan and colleagues (26) are represented in blue. The local maxima of the Fusiform Imagery Node (FIN) identified by a recent meta-analysis on visual mental imagery is represented in black. Coordinates of the axial and coronal slices are reported in MNI space.

To provide a more fine-grained anatomical description of the local maxima in the FFG that contribute to food-related processing, we overlaid the results of the current study on the brain map showing greater activity during food imagery as compared to water (**Figure 3**). We also overlaid the results of our previous study (28), and of previous literature on visual food processing (26), visual imagery (31), and trait food craving (17) (**Figure 3**, colored spheres). The spheres have a radius of 5 mm, they are centered at the MNI coordinates reported in the publication (**Figure 3**), and were created with the cluster and labelling module of the software Club (39). Only peaks in the left hemisphere were represented.

All the centroids of the spheres fell within the areas showing increased activation for food compared to water. Anatomically, the centroids were located in the posterior part of the occipitotemporal cortex, comprising the inferior temporal and fusiform gyri, and they were distributed along a lateral-medial axis.

At the descriptive level, the local maxima of the current study and of task-based neuroimaging data (26) were located more laterally, in the vicinity of the Fusiform Imagery Node (31), whereas the local maxima of resting-state data were positioned more medially in the occipitotemporal cortex (17, 28).

The present data suggest that the precise location of the local maxima observed in the current study is in line with previous neuroimaging evidence on the topic and hints at the possibility that food craving may be encoded by a defined portion of the occipitotemporal cortex.

#### 3.3.4 Influence of imagery performance on functional connectivity results

To rule out the influence of imagery quality on the degree of association between VTA-FFG functional connectivity and food craving, we performed non-parametric (Spearman) partial correlations. The correlation between differences in VTA-FFoA functional connectivity ([HC > W_HC_] > [LC > W_LC_]) and FCQ-T scores remained significant after controlling for imagery quality for HC, LC foods, and water (rho_(23)_=.76, p <.001). Similar results were obtained when additionally controlling for hunger, state food craving (FCQ-S scores), thirst, and state and trait anxiety as assessed by the state-trait anxiety inventory (STAI-X) (40) (rho_(23)_=.59, p=.01).

## 4 Discussion

Imagery of one’s own favorite food can trigger food cravings leading, in predisposed individuals, to subsequent overeating and weight gain. We tested the hypothesis that individual differences in food craving may be associated with the degree of functional coupling between the ventral tegmental area and the occipitotemporal cortex, particularly when prompted to imagine eating one’s own favorite high-calorie food.

### 4.1 Neural correlates of food cue-induced imagery

Food imagery was associated with the recruitment of a large brain network including regions typically associated with mental motor imagery, and sensory and reward-related brain areas specifically involved in (visual) food stimuli perception.

This fronto-parietal network is largely consistent with a “core” network involved in mental imagery tasks across different modalities (31, 32). When considering fMRI studies on imagery in different sensory modalities in a single meta-analysis, McNorgan observed significant convergent activity in a left-lateralized network comprising the prefrontal cortex, the parietal, and the occipital lobe (32). A more stringent conjunction analysis revealed a brain network restricted to the medial superior prefrontal cortex, the left inferior frontal gyrus, and the left inferior and superior parietal cortex (32), much in line with the brain network reported in the current study.

Additionally, we observed a cluster in the left insula involved in the imagery of both food and water. Early neurocognitive models of gustatory imagery suggest that taste perception and imagery converge in the insular cortex, with a predominant left activation during imagery^53^, and that the prefrontal cortex participates in the *“gustatory hallucinations”* by retrieving taste information from the insula (41, 42). Using multivariate pattern analysis, Avery and colleagues showed that neural activity of the mid-posterior insula in response to food pictures can reliably discriminate the dominant taste (sweet, sour, or salty) of the picture (43). During imagery of different foods, insula activity can reliably classify their primary taste qualities and those automatically inferred by viewing food pictures (cross-decoding) (44), suggesting that the insula holds multimodal taste information that is processed both via “bottom-up” sensory and “top-down” control processes.

Further, evoking mental images associated with the multisensory experience of eating food elicited the activity of a bilateral network comprising primary visual and somatosensory areas, the parietal cortex, and the prefrontal cortex, including the superior medial frontal cortex, the precentral gyrus, and the OFC. As expected, cue-triggered imagery of foods compared to water induced significant activation of two large bilateral clusters in the ventral occipitotemporal cortex, including the posterior portion of the inferior temporal gyrus and the FFG. Discrete cortical patches of the FFG respond selectively to food pictures (18–20), and activity of the ventral occipitotemporal cortex can classify with reliable accuracy the primary taste quality of imagined food and perceived food pictures (43, 44). The OFC receives information from all the sensory channels, and it is involved in representing and monitoring the value of biological and non-biological rewards (45, 46). Further, OFC activity is able to decode taste information in response to food pictures, but not in response to food imagery (44), suggesting that the bilateral OFC activity observed here may be related to the visual exposure to food pictures during the imagery task. Significant greater activity during food imagery compared to water was also observed in the precentral gyrus and in the right postcentral gyrus.

Taken together, task activation data suggest that participants were actively engaged in mental imagery, whereby a “core” fronto-parietal network modulates the activity of visual, gustatory, motor, and somatosensory areas to build a multisensory mental representation of food.

### 4.2 Trait food craving predicts functional coupling between the dopaminergic midbrain and higher-order visual regions

We recently observed altered resting state functional connectivity of the VTA in a sample of patients with obesity, expressed as greater connectivity with the putative FFoA and lower connectivity with the left inferior frontal gyrus (28). Across the whole sample, the degree of functional connectivity strength between the VTA and the left FFoA was positively associated with trait food craving and with the implicit bias for food stimuli, pointing to the possibility that VTA-FFoA functional coupling reflects the association between the visual representations of food and its rewarding and motivational properties (28).

In the current study, trait food craving was positively associated with the degree of functional connectivity between the VTA and a well-delimited cluster in the left FFG, such that higher food craving was linked to greater differences in VTA-FFG connectivity during imagery of liked high-calorie (HC) compared to disliked low-calorie (LC) foods. It is worth of notice that we did not use a ROI-based approach, rather we explored the entire brain using a canonical correction for multiple comparison on the data. The local maxima of the putative FFoA identified in the current study are located more laterally compared to the medial ones showing enhanced altered resting-state functional connectivity in obesity (28), yet very close to the local maxima reported by an Activation Likelihood Estimation meta-analysis of brain areas responsive to food (26). Conversely, the location of the medial FFoA maps closely to the one observed by Chen and colleagues (17) whose degree of resting-state local synchronization correlates positively with food craving. The present findings align with those of a recent meta-analysis on the neural correlates of visual mental imagery, whereby the authors identified a delimited region of the left lateral FFG (Fusiform Imagery Node) (31) as a key node of a visual imagery network, and proposed that fronto-parietal regions induce and modulate the activity of this network, contributing to the evocation and maintenance of visual mental images. Interestingly, the left-lateralized connectivity of the VTA with the FFoA confirms the results of our previous study and sustains the view that the left-hemisphere is chiefly involved in visual imagery (31), and may be preferentially activated by the perception of food stimuli (18).

Mechanistically, reward-related signals from midbrain dopaminergic nuclei to visual sensory cortices may lead to the establishment and/or maintenance of Pavlovian cue-reward associations. In mice, repeated pairings between visual stimuli and reward induce plastic changes in the neuronal representation of cues in the primary visual cortex, suggesting that external stimuli can modulate visual representations of cues early in the hierarchy of visual processing (47). In primates, electrical microstimulation of the VTA paired with visual stimulation enhances visual perceptual learning, and this effect is accompanied by fMRI signal changes in early visual, posterior inferotemporal, and anterior temporal regions (48, 49).

As a matter of speculation, VTA-FFoA connectivity may contribute to trait food craving by “tagging” with motivational nuances the food representations held in the FFoA, and greater VTA- FFoA functional synchronization during imagery of liked HC food may reflect stronger or more stable associations with its rewarding properties in people with high trait food craving. With this respect, VTA-FFoA connectivity may represent a core mechanism underlying food craving: patients with obesity are expected to show increased functional coupling not because of their BMI *per se*, but in virtue of their increased food cravings. Importantly, craving-related connectivity was not associated with state measures of craving, hunger, thirst, and quality of imagery suggesting that it is specifically related to stable and trait-like aspects of food craving.

Less expectedly, trait food craving was not associated with functional connectivity of the VTA with key targets of its dopaminergic projections, such as the nucleus accumbens and the amygdala. Further, we could not find evidence for a significant negative association between trait food craving and connectivity of the VTA with the prefrontal cortex (PFC). Different explanations may account for these negative findings. A study by Pimpini and colleagues showed that the level of activity of the brain reward circuitry in response to food cues is higher when participants focus on its hedonic (i.e., tastiness) as compared to its health (i.e., caloric content) and perceptual (i.e., color) features (50). In our study, participants were instructed to focus on the multisensory experience of eating food, comprising its taste, smell, and texture in the mouth: as a consequence, it is likely that participants’ focus of attention was not consistent within and between participants, leading to heterogeneous responses of the brain reward circuitry. The association between meso-striatal connectivity and trait food craving during imagery of palatable HC foods may thus be dependent upon the participants’ focus of attention: being present or stronger when focusing on the hedonic properties of food, and absent or weaker when focusing on other characteristics of food, such as caloric content or perceptual features. In a previous study reporting a positive correlation between striatal responses to HC foods and trait food craving, participants’ weight status ranged from normal weight to obesity, suggesting that the association may emerge once considering trait food craving in people with past or current history of overeating (16). Similarly, the lack of association between trait food craving and VTA-PFC connectivity may be partly due to the selection of healthy-weight participants with no current or prior eating-related disorders or obesity, for which food craving is not accompanied by reduced inhibitory control.

## 5 Conclusions

The present findings highlight the role of functional connectivity between the dopaminergic midbrain and higher-order visual regions in food craving. We showed that VTA-FFG connectivity is associated with trait food craving, specifically during exposure and imagery of eating liked HC foods, suggesting a neurofunctional mechanism by which the mental representations of the food we like can become so salient and irresistible. This further evidence suggests that people with higher trait food craving may benefit the most from cognitive-behavioral interventions targeting cue-reward associations. Future studies should determine the potential of such interventions to “down-regulate” the connectivity between the dopaminergic midbrain and higher-order visual areas, thus modulating the desire for the perceived or imagined food.

## Data availability statement

The raw data supporting the conclusions of this article will be made available by the authors, without undue reservation.

## Ethics statement

The studies involving humans were approved by IRCCS San Raffaele of Milan; Prot. CONSUME, 58/INT/2020. The studies were conducted in accordance with the local legislation and institutional requirements. The participants provided their written informed consent to participate in this study.

## Author contributions

FD: Data curation, Formal analysis, Methodology, Software, Visualization, Writing – original draft, Writing – review & editing. MM: Conceptualization, Data curation, Formal analysis, Investigation, Methodology, Software, Visualization, Writing – original draft, Writing – review & editing. EG: Data curation, Formal analysis, Investigation, Methodology, Software, Writing – original draft, Writing – review & editing. EP: Methodology, Supervision, Visualization, Writing – original draft, Writing – review & editing. LZ: Conceptualization, Data curation, Formal analysis, Funding acquisition, Investigation, Methodology, Project administration, Resources, Software, Supervision, Validation, Visualization, Writing – original draft, Writing – review & editing.
