# Supplementary material for: Trait food craving predicts functional connectivity between dopaminergic midbrain and the fusiform food area during eating imagery
Source: Front Psychiatry. 2024 May 7;15:1396376. doi: 10.3389/fpsyt.2024.1396376 (PMC11107427; doi:10.3389/fpsyt.2024.1396376)
Supplement: Supplementary file 1 [file DataSheet_1.pdf]

Supplementary Materials

Trait food craving predicts functional connectivity between the dopaminergic midbrain and the fusiform food area during eating imagery

Francantonio Devoto[1*†], Marika Mariano[1†], Edoardo Gornetti[1],
Eraldo Paulesu[1,2] & Laura Zapparoli[1,2*]

[1] Psychology Department and NeuroMi – Milan Centre for Neuroscience, University of Milano-Bicocca, Milan, Italy
[2] fMRI Unit, IRCCS Orthopedic Institute Galeazzi, Milan, Italy

*** Corresponding authors**
Francantonio Devoto & Laura Zapparoli
Psychology Department
University of Milano-Bicocca
Email: francantonio.devoto@unimib.it; laura.zapparoli@unimib.it

[†] **These authors contributed equally to this work and share the first authorship.**

Index

1. Supplementary methods *3*
1.1. Participants **3**
1.2. Behavioral measures **3**
1.3. Food cue-reactivity imagery paradigm **3**
1.4. Task functional activation analyses **4**
1.5. Task functional connectivity analyses **5**
2. Supplementary Tables *7*
2.1. Supplementary Table 1 .... **7**
2.2. Supplementary Table 2 .... **8**
2.3. Supplementary Table 3 .... **12**
3. Supplementary figures *15*
3.1. Supplementary Figure 1 .... **15**
3.2. Supplementary Figure 2 .... **16**
4. References *17*

1. Supplementary methods

1.1. Participants

Participants were required to fast for 4-5 hours prior to the experiment and to avoid drinking beverages other than water. Upon their arrival, all participants completed a screening for eating disorders, including anorexia nervosa, bulimia nervosa, and binge-eating disorder.

In particular, participants were presented with: (i) the Binge Eating Scale (BES)[1], a measure constructed to described behavioral manifestations and feelings/cognitions surrounding a binge episode; and (ii) the Eating Disorder Inventory (EDI-3)[2], a multiscale measure designed for the assessment of psychological and behavioral traits common in anorexia nervosa. As regards this last scale, we considered the following subscales: Drive for Thinness, Body Dissatisfaction, and Bulimia. Based on these scales, we were able to identify and exclude one participant with pathological scores. Then, their height and weight were measured to allow the calculation of the BMI. Participants' demographic information is reported in Table S1.

1.2. Behavioral measures

Food preferences

Individual food preferences were assessed online by asking participants to report their liking rating on a 6-point Likert scale ranging from 0 (I don't like it at all) to 6 (I extremely like it) for 100 foods (50 HC and 50 LC foods). Food stimuli were taken from the Food-pics database, a standardized and validated database of food pictures differing in caloric and macronutrient content[3]. HC food stimuli included caloric-dense and fat foods such as pizza, hamburger, and chocolate snacks, whereas LC foods mainly consisted of vegetables (e.g., spinaches, salad, carrots) and low-fat foods rich in proteins (e.g., fish, grilled chicken meat).

Six of the most liked HC foods and six of the least liked LC foods were chosen as stimuli for each participant (see Table S2).

Food craving

Food craving was assessed by means of the Food Craving Questionnaire - Trait (FCQ-T)[4,5], a self-report measure evaluating food craving in general. The questionnaire has 39 items that can lead to a total score (higher total score, higher trait food cravings) and to several subscales scores. Prior to fMRI, food craving was assessed by means of the Food Craving Questionnaire - State (FCQ-S)[4,5], a self-report measure that evaluates the current food craving. The questionnaire has 15 items that can lead to a total score (higher total score, higher state food cravings) and to several subscales scores.

Hunger and thirst

Prior to the fMRI scanning, hunger was assessed by means of a 100-mm visual analogue scale (VAS) ranging from 0 (not hungry at all) to 100 (extremely hungry).

Thirst was assessed by means of a 100-mm VAS ranging from 0 (not thirsty at all) to 100 (extremely thirsty, see Figure S1A in Supplementary Information).

1.3. Food cue-reactivity imagery paradigm

fMRI scanning was performed during a food cue-reactivity paradigm involving visually guided mental imagery. Participants were instructed to imagine eating the food (liked HC and least liked LC food) or to imagine drinking a glass of water, depending on the picture displayed on the screen. Subjects were instructed to focus on the multisensory experience of eating the food, including its smell, taste, and texture experienced in the mouth. At the end of the fMRI task, participants were asked to rate the quality of their imagery performance separately for HC, LC foods and water by means of 100-mm VAS ranging from 0 (extremely bad) to 100 (extremely good; see Figure S1A).

The task included 4 blocks: (i) HC food imagery, (ii) control water imagery (after HC food), (iii) LC food imagery, (iv) control water imagery (after LC food). Each block for food (HC, LC) and water (after HC, after

LC) imagery lasted 18 s, during which participants were asked to perform mental imagery while the food/water picture remained on the screen. After each block, a blank screen with the string "stop imagining" was displayed for 2 s (rest period).

Trials followed a fixed order, whereby a HC imagery block (HC imagery - water imagery) was followed by a LC imagery block (LC imagery - water imagery). The HC and LC imagery blocks were repeated 6 times. The water picture represented a glass of water (W; see Figure S1B).

Since data of self-report imagery quality significantly deviated from normal distribution, differences in imagery quality across the three conditions (HC, LC, W) were evaluated by means of a non-parametric repeated measures ANOVA (Friedman test). We also employed Bonferroni-corrected Wilcoxon signed-rank tests were performed to test for pairwise differences in imagery quality. Differences in imagery quality between food (average of HC and LC) and water were tested with a paired-samples t-test, as data did not deviate significantly from the normal distribution. Results were deemed significant with $p < .05$. The analyses were performed in R, using the "rstatix" package.

1.4. Task functional activation analyses

Preprocessing

After image reconstruction, raw data visualization and conversion from DICOM to the NIFTI format were performed with MRIcron (www.mricro.com) software. All subsequent data analyses were performed in MATLAB R2016b (Mathworks. Natick. MA. USA) using the software Statistical Parametric Mapping (SPM12, Wellcome Department of Imaging Neuroscience, London, UK).

Functional data were realigned using SPM realign & unwarp procedure, where all scans were coregistered to a reference image (first scan of the run) using a least squares approach and a 6 parameter (rigid body) transformation and resampled using b-spline interpolation to correct for motion and magnetic susceptibility interactions. Potential outlier scans were identified using ART as acquisitions with framewise displacement above 0.9 mm or global BOLD signal changes above 5 standard deviations. Two experimental subjects exhibited more than 20% outlier scans in the whole experimental run and were excluded from the subsequent statistical analyses, for a final sample of 25 participants.

Functional and anatomical data were normalized into standard MNI space, segmented into grey matter, white matter, and CSF tissue classes, and resampled to 2 mm isotropic voxels following a direct normalization procedure using SPM unified segmentation and normalization algorithm[6,7].

Last, the stereotactically normalized scans were smoothed using a Gaussian filter of 10 x 10 x 10 mm to improve the signal- to-noise ratio, making the data suited for cluster level correction for multiple comparisons[8].

First level analyses

Individual scans were weighted by a boxcar signal characterizing each individual experimental condition (i.e., imagine HC, imagine W_{HC}, imagine LC, imagine W_{LC}, rest) convolved with an SPM canonical hemodynamic response function and rectified[9].

Global differences in the fMRI signal were removed from all voxels with proportional scaling. High-pass filtering (128 s) was used to remove artefactual contributions to the fMRI signal, such as physiological noise from cardiac or respiratory cycles. A fixed-effect analysis was performed for each subject to characterize the BOLD response associated with the task before entering the relevant individual contrast images into a random-effect analysis.

At the first level, we characterized the brain activity recorded between the appearance and disappearance of the food or water's pictures, namely the entire imagery process. We included one regressor for each condition (HC food, water after HC food, LC food, water after LC food, rest) for a total of five regressors. The parameters obtained from the realignment and outlier detection procedure were added as non-interest regressors as well to partial out the impact of motion artifacts and outlier scans on the estimates of the beta parameters.

For each participant, we considered an implicit baseline leading to four contrast images: (i) HC food, (ii) LC food, (ii) water after HC food (W_{HC}), and (iv) water after LC food (W_{LC}).

Second level analyses

In a second-level full-factorial random-effect analysis, two contrasts were computed in order to (i) identify the neural networks commonly involved in mental imagery of food and water (food ∩ water) and (ii) distinguish the brain areas more active during the imagery of food compared to water (food > water):

i. Conjunction effect of food (HC,LC) and water (W_{HC},W_{LC}) imagery [FOOD ∩ WATER]. The conjunction effect was tested to identify the neural networks commonly involved in mental imager of food and water.

ii. Effect of food (HC,LC) over water (W_{HC},W_{LC}) imagery [FOOD > WATER]. This contrast was performed to distinguish the brain areas more active during the imagery of food (HC and LC) compared to water (baseline condition).

Cluster-level inferences were based on parametric statistics from Gaussian Random Field theory[10]. Results were thresholded using a combination of a voxel-level $p < 0.001$ uncorrected threshold, and a familywise corrected p-FWE < 0.05 cluster-size threshold across the whole brain[8].

1.5. Task functional connectivity analyses

Preprocessing and denoising

After image reconstruction, raw data visualization and conversion from DICOM to the NIFTI format were performed with MRIcron (www.mricro.com) software. Data analyses were performed in MATLAB R2016b (Mathworks. Natick. MA. USA) using the software CONN[11] and SPM12.

Functional data were realigned using SPM realign & unwarp procedure, where all scans were coregistered to a reference image (first scan of the run) using a least squares approach and a 6 parameter (rigid body) transformation and resampled using b-spline interpolation to correct for motion and magnetic susceptibility interactions. Temporal misalignment between different slices of the functional data was corrected following SPM slice-timing correction procedure, using sinc temporal interpolation to resample each slice BOLD timeseries to a common mid-acquisition time.

Potential outlier scans were identified using ART as acquisitions with framewise displacement above 0.9 mm or global BOLD signal changes above 5 standard deviations. Two experimental subjects exhibited more than 20% outlier scans in the whole experimental run and were excluded from the subsequent statistical analyses (the same two participants excluded in the previous task-based fMRI analysis).

Functional and anatomical data were normalized into standard MNI space, segmented into grey matter, white matter, and CSF tissue classes, and resampled to 2 mm isotropic voxels following a direct normalization procedure using SPM unified segmentation and normalization algorithm[6,7].

Last, the stereotactically normalized scans were smoothed using a Gaussian filter of 10 x 10 x 10 mm to improve the signal-to-noise ratio, making the data suited for cluster-level correction for multiple comparisons[8].

In addition, functional data were denoised by including the regression of potential confounding effects characterized by white matter timeseries (5 CompCor noise components), CSF timeseries (5 CompCor noise components), motion parameters and their first order derivatives (12 factors), outlier scans, session and task effects (imagine HC, imagine W_{HC}, imagine LC, imagine W_{LC}, rest) and their first order derivatives, and linear trends, followed by high-pass frequency filtering of the BOLD timeseries above 0.008 Hz. CompCor[12,13] noise components within white matter and CSF were estimated by computing the average BOLD signal as well as the largest principal components orthogonal to the BOLD average, motion parameters, and outlier scans within each subject's eroded segmentation masks.

First level analyses

Seed-based functional connectivity maps were estimated characterizing the spatial pattern of functional connectivity with the seed area, as defined by the bilateral VTA of the AAL3 template[14].

Functional connectivity strength was represented by Fisher-transformed bivariate correlation coefficients from a weighted general linear model (weighted-GLM), estimated separately for each seed area and target voxel, modeling the association between their BOLD signal timeseries.

Individual scans were weighted by a boxcar signal characterizing each individual experimental condition (i.e., imagine HC, imagine W_{HC}, imagine LC, imagine W_{LC}, rest) convolved with an SPM canonical hemodynamic response function and rectified.

Second level analyses

Group-level analyses were performed using a General Linear Model (GLM) conforming to a 2 x 2 repeated-measures ANOVA, with food (HC vs. LC) and water (W_{HC} vs. W_{LC}) as within-subjects factors. For each individual voxel a separate GLM was estimated, with first-level connectivity measures at this voxel as dependent variables (one independent sample per subject and one measurement per experimental condition), and total trait food craving scores as independent variable. Voxel-level hypotheses were evaluated using multivariate parametric statistics with random-effects across subjects and sample covariance estimation across multiple measurements. Inferences were performed at the level of individual clusters (groups of contiguous voxels).

Linear regressions were performed to test whether trait food craving scores were associated with differences in whole-brain functional connectivity of the VTA during the following contrasts (food-by-water interactions):

i. [HC > W_{HC}] > [LC > W_{LC}]. To identify the brain areas whose connectivity with the VTA during imagery of HC compared to LC foods is positively associated with trait food craving scores;
ii. [LC > W_{LC}] > [HC > W_{HC}]. To identify the brain areas whose connectivity with the VTA during imagery of LC compared to HC foods is positively associated with trait food craving scores.

Cluster-level inferences were based on parametric statistics from Gaussian Random Field theory[10]. Results were thresholded using a combination of a voxel-level $p < 0.001$ uncorrected threshold, and a familywise corrected p-FWE < 0.05 cluster-size threshold across the whole brain[8].

2. Supplementary Tables

2.1. Supplementary Table 1

Table S1 | Demographic information. Participants' demographic information. Means (standard deviations) are shown. BMI, body-mass index.

	**N = 28**
**Gender (M/F)**	15/13
**Education (years)**	15 (2.1)
**Age (years)**	24 (4.1)
**BMI (kg/m^2)**	21.2 (2)
**Fasting from test (minutes)**	299.5 (49.2)

2.2. Supplementary Table 2

Table S2. Stimuli selection for the food cue-reactivity imagery task | The most liked high-calorie and the least liked low-calorie food stimuli selected for each participant are reported. For both low and high-calorie food, the identification number of images from the Food-pics[3] database, the calories of the food, and the liking, wanting, and frequency scores are listed.

ID	#Image	Kcal	Liking	#Image	Kcal	Liking
	LC			HC		
ID01	261	42	1	184	729	5
	257	63	2	56	330	5
	251	143	2	127	244	6
	267	20	2	489	858	6
	258	5	2	176	913	5
	444	34	2	514	511	5
ID02	382	4	1	175	548	4
	424	109	2	163	451	4
	262	33	2	559	972	5
	434	54	2	416	362	6
	272	8	2	127	244	6
	258	5	2	539	478	6
ID03	435	139	1	2	907	6
	382	4	1	101	255	6
	442	63	1	189	345	6
	273	18	1	19	951	6
	356	214	1	184	729	6
	368	105	1	286	530	6
ID04	401	4	1	111	555	6
	545	5	1	189	345	6
	260	18	1	289	259	6
	251	143	2	176	913	6
	262	33	2	19	951	6
	382	4	2	286	530	6
ID05	262	33	1	22	649	6
	362	30	1	83	301	6
	273	18	1	45	580	6
	368	105	1	87	505	6
	502	16	1	53	267	6
	329	97	1	176	913	6
ID06	442	63	1	289	259	6
	482	47	1	167	530	6
	445	51	1	184	729	6
	434	54	1	163	451	6
	272	8	1	87	505	6
	444	34	1	111	555	6
ID07	520	12	1	184	729	6
	257	63	2	163	451	6

	382	4	2	175	548	6
	273	18	2	22	649	6
	434	54	2	556	609	6
	258	5	3	410	1683	6
ID08	257	63	1	290	1626	5
	257	63	1	556	609	5
	360	55	1	59	2844	5
	446	14	1	2	907	5
	545	5	1	19	951	6
	263	14	1	22	649	5
ID09	401	4	1	289	259	5
	382	4	1	127	244	6
	362	30	1	287	260	6
	273	18	1	111	555	6
	368	105	1	2	907	5
	432	35	1	318	489	5
ID10	251	143	1	87	505	6
	262	33	1	53	267	6
	435	139	1	27	1070	6
	303	12	1	286	530	6
	263	14	1	157	340	6
	368	105	1	22	649	6
ID11	257	63	1	286	530	6
	435	139	1	403	858	6
	360	55	1	56	330	6
	303	12	1	68	672	6
	272	8	1	19	951	6
	267	20	1	176	913	6
ID12	262	33	1	6	396	6
	273	18	1	2	907	6
	435	139	2	83	301	6
	520	12	2	101	255	6
	257	63	2	87	505	6
	251	143	2	556	609	6
ID13	272	8	1	184	729	5
	251	143	2	127	244	6
	442	63	2	6	396	6
	368	105	2	2	907	6
	438	17	2	19	951	6
	260	18	2	559	972	5
ID14	446	14	1	157	340	5
	273	18	1	416	362	6
	303	12	1	176	913	4
	267	20	1	556	609	4
	482	47	1	189	345	4
	277	30	1	53	267	4

ID15	262	33	1	2	907	5
	435	139	1	27	1070	5
	360	55	1	176	913	5
	303	12	1	516	623	5
	368	105	1	6	396	5
	502	16	1	489	858	5
ID16	257	63	1	410	1683	6
	273	18	1	515	766	5
	401	4	2	184	729	6
	267	20	2	176	913	5
	395	209	2	527	416	5
	258	5	2	286	530	5
ID17	262	33	1	6	396	5
	435	139	1	559	972	5
	360	55	1	2	907	5
	303	12	1	27	1070	5
	260	18	1	176	913	5
	250	85	1	516	623	5
ID18	262	33	2	6	396	6
	368	105	2	163	451	6
	502	16	2	68	672	6
	267	20	2	150	238	5
	258	5	2	287	260	6
	361	39	2	175	548	6
ID19	502	16	1	19	951	6
	266	44	1	150	238	6
	335	9	1	22	649	6
	361	39	1	2	907	6
	434	54	2	101	255	6
	272	8	2	151	653	6
ID20	401	4	1	27	1070	5
	264	4	1	516	623	6
	360	55	1	59	2844	6
	303	12	1	416	362	6
	356	214	1	2	907	6
	263	14	1	87	505	6
ID21	257	63	1	27	1070	6
	251	143	1	167	530	6
	262	33	1	290	1626	6
	435	139	1	176	913	6
	446	14	1	19	951	6
	382	4	1	150	238	6
ID22	424	109	2	111	555	6
	267	20	2	189	345	6
	444	34	2	53	267	6
	262	33	3	160	243	6

	435	139	3	68	672	6
	360	55	3	19	951	6
ID23	360	55	1	352	2205	5
	442	63	1	68	672	6
	303	12	1	167	530	6
	424	109	1	189	345	6
	368	105	1	515	766	5
	267	20	1	127	244	6
ID24	257	63	1	403	858	6
	251	143	1	12	536	6
	262	33	1	27	1070	6
	362	30	1	167	530	6
	303	12	1	416	362	6
	267	20	1	556	609	5
ID25	434	54	1	87	505	3
	272	8	1	289	259	3
	266	44	1	176	913	2
	401	4	2	556	609	2
	435	139	2	184	729	3
	321	77	2	286	530	3
ID26	435	139	1	489	858	6
	382	4	1	556	609	6
	362	30	1	116	270	6
	356	214	1	127	244	6
	263	14	1	410	1683	6
	502	16	1	559	972	6
ID27	424	109	1	489	858	6
	356	214	1	403	858	6
	277	30	1	27	1070	5
	262	33	2	176	913	5
	401	4	2	559	972	6
	435	139	2	111	555	5
ID28	257	63	1	159	244	6
	435	139	1	19	951	6
	360	55	1	56	330	6
	272	8	1	352	2205	6
	258	5	1	150	238	5
	261	42	1	127	244	6

2.3. Supplementary Table 3

Table S3 | fMRI results for the task activation analyses | A. Results of the conjunction analysis: brain regions significantly active during imagery of food stimuli and water. **B.** Results of the contrast analysis: brain regions significantly more active during imagery of food compared to water. For each region, we reported the anatomical labelling according to the AAL3 template[14] (Brodmann area), the coordinates in MNI space, and the Z-score. *, peak-level FWE-corrected.

	Left hemisphere				Right hemisphere			
**Anatomical label (Brodmann area)**	**X**	**Y**	**Z**	**Z-score**	**X**	**Y**	**Z**	**Z-score**
**A. Conjunction analysis**								
Superior Frontal gyrus (32)	-12	20	44	3.95				
Middle Frontal gyrus (45)	-42	30	30	4.00				
Inferior Frontal gyrus, opercular part (44)	-50	10	0	4.88*				
	-50	8	20	4.56*				
Inferior Frontal gyrus, triangular part (45,48)	-42	40	10	4.51				
	-42	36	24	4.04				
	-40	36	14	4.46				
Supplementary motor area (6, 32)					6	12	58	4.61*
	-8	14	44	3.55				
	-2	10	62	3.94				
	-2	14	54	3.96				
Inferior Parietal gyrus (40)	-56	-40	50	5.09*				
	-54	-42	46	5.12*				
	-50	-44	52	4.95*				
Insula (48)	-46	10	-2	5.01*				
	-38	18	8	3.71				
	-36	8	8	3.89				
**B. Food > Water**								

Lateral orbital gyrus (47)	-42	36	-16	5.811*				
Posterior orbital gyrus (11, 47)	-32	34	-16	5.67*				
					24	24	-22	4.20
Anterior orbital gyrus (11)					26	36	-20	5.57*
					20	48	-16	3.81
Superior Frontal gyrus (6, 8, 9)					34	0	68	3.56
					28	4	70	3.46
					20	20	66	3.27
	-30	-4	66	3.71				
	-28	44	40	3.44				
Middle Frontal gyrus (6, 8, 46)					48	0	54	3.89
	-40	36	36	3.90				
	-30	12	62	3.43				
Inferior frontal gyrus, triangular part (45, 48)	-42	24	24	5.52*				
	-40	34	16	6.41*				
Supplementary motor area (6)	-14	12	66	4.16				
	-8	10	72	4.06				
	-2	16	48	5.99*				
Precentral gyrus (6, 44)					60	2	38	5.95*
					56	4	44	5.30*
	-56	8	34	7.13*				
	-54	8	40	7.07*				
	-46	4	30	6.81*				
Postcentral gyrus (2, 3, 43)					64	-14	32	7.42*
					58	-24	50	4.10
					46	-40	62	4.08
					44	-30	38	3.56

	-60	-20	36	6.11*				
Supramarginal gyrus (2)	-58	-26	42	6.00*				
Superior Parietal gyrus (7)					28	-60	56	6.23*
	-24	-54	48	7.47*				
	-22	-64	50	7.7*				
	-22	-68	40	7.47*				
	-20	-70	52	7.74*				
Inferior Parietal gyrus (2, 40)	-44	-34	40	6.90*				
	-44	-42	56	4.78*				
Superior Occipital gyrus (18, 19)					28	-68	34	5.99*
	-22	-68	34	7.64*				
Middle Occipital gyrus (17, 18)	-28	-92	-4	>8*				
	-26	-96	0	>8*				
	-24	-100	2	>8*				
Inferior Occipital gyrus (18, 37)					36	-92	-4	>8*
					24	-94	-2	>8*
	-38	-66	-8	>8*				
	-22	-90	-10	>8*				
Inferior Temporal gyrus (37)					44	-64	-12	6.90*
Fusiform gyrus (19, 37)					40	-62	-20	6.43*
					38	-58	-20	6.34*
Cerebellum (37)	-28	-52	-18	6.45*				

3. Supplementary figures

3.1. Supplementary Figure 1

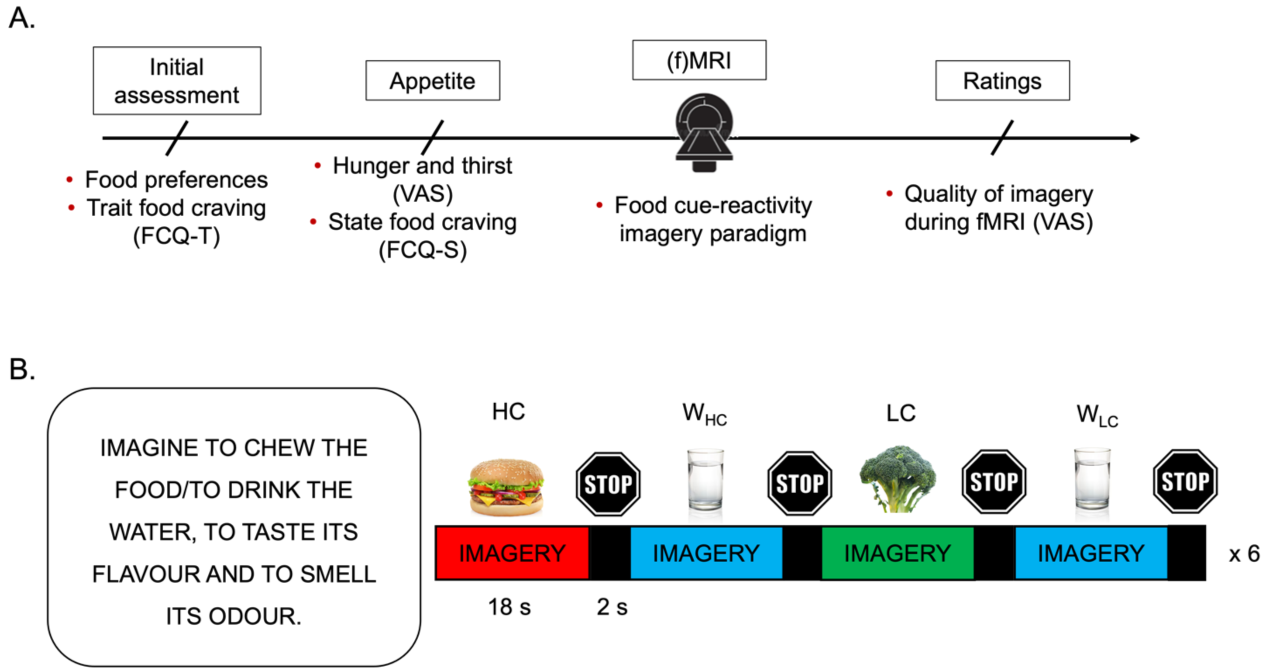

Figure S1 | Procedure and task structure. A. Procedures of the study. **B.** Structure of the food cue-reactivity imagery task. HC, high-calories food; LC, low-calories food; FCQ-T, Food Cravings Questionnaire trait; FCQ-S, Food Cravings Questionnaire state; VAS, visual analogue scale; W, water.

3.2. Supplementary Figure 2

Figure S2 | Results for the quality of imagery ratings. HC, high-calories food; LC, low-calories food; W, water. ** p < .01, *** p < .001.

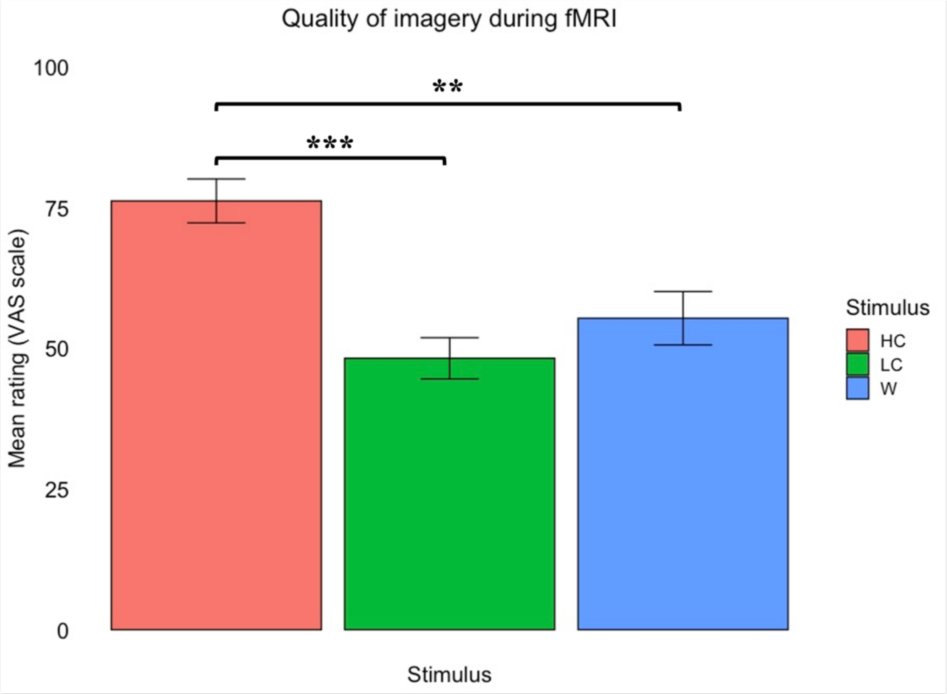

4. References

1. Gormally J, Black S, Daston S, Rardin D. The assessment of binge eating severity among obese persons. *Addict Behav*. 1982;7(1):47-55. doi:10.1016/0306-4603(82)90024-7
2. Garner DM. *Eating disorder inventory-3 (EDI-3)*. vol*: Psychological Assessment Resources . Professional manual* 2004.
3. Blechert J, Meule A, Busch NA, Ohla K. Food-pics: an image database for experimental research on eating and appetite. *Front Psychol*. 2014;5:617. doi:10.3389/fpsyg.2014.00617
4. Cepeda-Benito A, Gleaves DH, Fernández MC, Vila J, Williams TL, Reynoso J. The development and validation of Spanish versions of the State and Trait Food Cravings Questionnaires. *Behav Res Ther*. Nov 2000;38(11):1125-38. doi:10.1016/s0005-7967(99)00141-2
5. Innamorati M, Imperatori C, Balsamo M, et al. Food Cravings Questionnaire-Trait (FCQ-T) discriminates between obese and overweight patients with and without binge eating tendencies: the Italian version of the FCQ-T. *J Pers Assess*. 2014;96(6):632-9. doi:10.1080/00223891.2014.909449
6. Ashburner J, Friston KJ. Unified segmentation. *Neuroimage*. Jul 01 2005;26(3):839-51. doi:10.1016/j.neuroimage.2005.02.018
7. Ashburner J. A fast diffeomorphic image registration algorithm. *Neuroimage*. Oct 15 2007;38(1):95-113. doi:10.1016/j.neuroimage.2007.07.007
8. Flandin G, Friston KJ. Analysis of family-wise error rates in statistical parametric mapping using random field theory. *Hum Brain Mapp*. 05 2019;40(7):2052-2054. doi:10.1002/hbm.23839
9. Worsley KJ, Friston KJ. Analysis of fMRI time-series revisited--again. *Neuroimage*. Sep 1995;2(3):173-81. doi:10.1006/nimg.1995.1023
10. Worsley KJ, Marrett S, Neelin P, Vandal AC, Friston KJ, Evans AC. A unified statistical approach for determining significant signals in images of cerebral activation. *Hum Brain Mapp*. 1996;4(1):58-73. doi:10.1002/(SICI)1097-0193(1996)4:1<58::AID-HBM4>3.0.CO;2-O
11. Whitfield-Gabrieli S, Nieto-Castanon A. Conn: a functional connectivity toolbox for correlated and anticorrelated brain networks. *Brain Connect*. 2012;2(3):125-41. doi:10.1089/brain.2012.0073
12. Behzadi Y, Restom K, Liau J, Liu TT. A component based noise correction method (CompCor) for BOLD and perfusion based fMRI. *Neuroimage*. Aug 2007;37(1):90-101. doi:10.1016/j.neuroimage.2007.04.042
13. Chai XJ, Castañón AN, Ongür D, Whitfield-Gabrieli S. Anticorrelations in resting state networks without global signal regression. *Neuroimage*. Jan 16 2012;59(2):1420-8. doi:10.1016/j.neuroimage.2011.08.048
14. Rolls ET, Huang CC, Lin CP, Feng J, Joliot M. Automated anatomical labelling atlas 3. *Neuroimage*. 02 01 2020;206:116189. doi:10.1016/j.neuroimage.2019.116189
